# Severe Neonatal Cholestasis as an Early Presentation of McCune- Albright Syndrome

**DOI:** 10.4274/jcrpe.galenos.2018.2018.0110

**Published:** 2019-02-20

**Authors:** Nicole Coles, Ian Comeau, Tatiana Munoz, Jennifer Harrington, Roberto Mendoza-Londono, Andreas Schulze, Sari Kives, Binita M. Kamath, Jill Hamilton

**Affiliations:** 1 University of Toronto, Hospital for Sick Children, Clinic of Endocrinology, Toronto, Canada; 2 Montreal Children’s Hospital, Clinic of Adolescent Medicine and Paediatric Gynaecology, Montreal, Canada; 3 University of Toronto, Hospital for Sick Children, Clinic of Clinical and Metabolic Genetics, Toronto, Canada; 4 University of Toronto, Hospital for Sick Children, Clinic of Paediatric Gynaecology, Toronto, Canada; 5 University of Toronto, Hospital for Sick Children, Clinic of Gastroenterology, Hepatology and Nutrition, Toronto, Canada

**Keywords:** McCune-Albright syndrome, neonatal cholestasis, precocious puberty

## Abstract

McCune-Albright syndrome (MAS) is a rare genetic disorder characterized by café-au-lait macules, polyostotic fibrous dysplasia and multiple endocrinopathies. Liver involvement, although described, is a rare complication. We review the case of a child with MAS whose initial presentation was characterized by severe neonatal cholestasis. The case demonstrates a severe phenotype of persistent cholestasis in MAS requiring liver transplantation. This phenotype has been previously considered to be a more benign feature. This case highlights the importance of consideration of MAS as an uncommon but important cause of neonatal cholestasis. Early diagnosis may allow for prompt recognition and treatment of other endocrinopathies.

**What is already known on this topic?**

Hepatic disease is a rare but described feature of McCune-Albright syndrome (MAS). Previous descriptions of cholestasis in MAS have detailed a benign phenotype with gradual improvement and resolution over time.

**What this study adds?**

This case demonstrates a presentation of severe cholestasis in McCune-Albright syndrome with significant associated morbidity, ultimately leading to liver transplantation.

## Introduction

Classically, McCune-Albright syndrome (MAS) is characterized by café-au-lait macules, polyostotic fibrous dysplasia and multiple endocrinopathies, including precocious puberty. The condition presents with different tissue involvement depending on the location of the postzygotic somatic mutations, resulting in wide phenotypic variability. Understanding the diverse manifestations of this rare diagnosis is important as it is associated with potentially significant complications including short stature, fractures, facial deformity and hearing and vision loss.

## Case Report

The index patient is a female infant born following an uncomplicated pregnancy. She was delivered at 40 weeks gestation with a birth weight of 2020 g and presented at two weeks of life with significant neonatal cholestasis. She had persistent jaundice and mild hepatomegaly with laboratory investigations showing evidence of conjugated hyperbilirubinemia [peak conjugated bilirubin 193 micromol/L, (normal range: 0-2 micromol/L)] and marked elevation of liver enzymes [peak aspartate aminotransferase (AST): 3633 U/L, (normal range: 0-110 U/L), peak alanine aminotransferase (ALT): 2120 U/L, (normal range: 0-60 U/L) and peak gamma-glutamyl transferase: 106, (normal range: 0-45 U/L)]. Further investigations revealed normal coagulation parameters [international normalized ratio: 0.9 (normal range: 0.6-1.6), partial thromboplastin time 38s (normal range: 25-44s)] and albumin levels [41g/L (normal range: 26-41 g/L)]. The patient underwent a comprehensive panel of investigations to determine the cause of the hepatobiliary dysfunction, but no clear diagnosis was identified. She had a normal TORCH screen, hepatitis B and C serologies, alpha-1-anti-trypsin level, galactosemia screen, metabolic and mitochondrial studies, urine bile acids and negative genetic testing for Niemann-Pick type C, Alagille syndrome and progressive familial intra-hepatic cholestasis. Imaging of the liver revealed mild hepatomegaly with non-specific mild heterogeneity and no evidence of biliary obstruction. A liver biopsy was performed at one month of age and showed significant liver damage with cytoplasmic and canalicular cholestasis, broad areas of resolving hepatocellular necrosis, giant cell transformation and abundant extramedullary hematopoiesis.

She experienced ongoing severe cholestasis with secondary complications, namely significant fat malabsorption and failure to thrive requiring placement of an enterostomy tube. The patient experienced episodes of recurrent respiratory infections and sepsis. She also sustained a non-traumatic femur fracture, which was thought to be related to metabolic bone disease in the setting of profound cholestasis and poor nutrition. As a result of these progressive complications, she was listed for liver transplantation and underwent a living related donor transplant at the age of 10 months. Histologic examination of the liver explant showed severe cholestatic liver disease with intrahepatic cholestasis, focal bile cannalicular plugs, mild to moderate focal peri-portal and sinusoidal fibrosis, yielding no definitive cause for the liver disease.

At 14 months of age, she presented with a five-day history of vaginal bleeding. She was otherwise well, with no history of fevers, discharge or abdominal pain. Physical examination was significant for sexual precocity including Tanner stage 2 breasts and pubic hair with an estrogenized vulva. Dermatological examination revealed multiple ragged-edge café-au-lait macules in the lumbosacral area and bilaterally on her legs.

**Figure 1:**
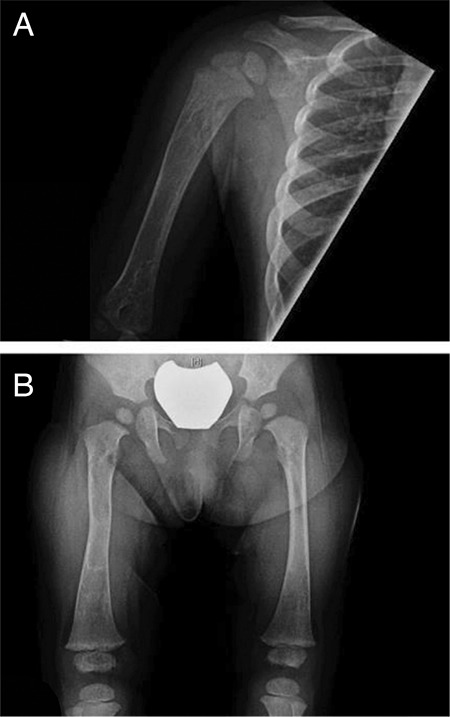
Skeletal survey demonstrating findings suggestive of polyostotic fibrous dysplasia. A) Ill-defined foci of linear sclerosis and lucency in the right proximal humerus. B) Illdefined lucency with associated sclerosis in the left proximal femoral shaft

**Figure 2:**
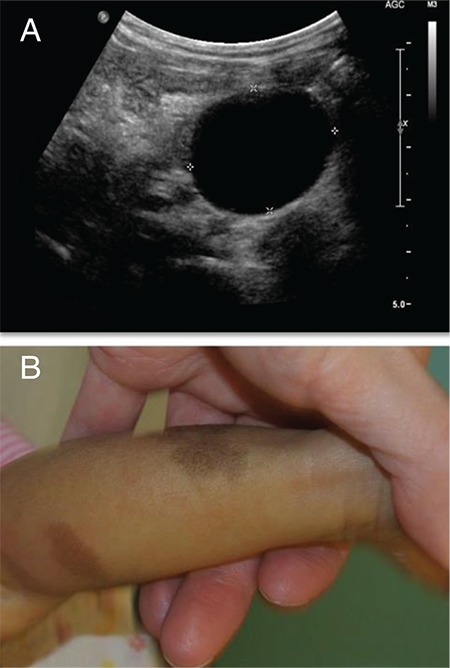
Clinical features suggestive of McCune-Albright syndrome. A) Abdominal ultrasound demonstrating large cystic structure in left ovary. B) Café-au-lait macules present bilaterally on legs and lumbosacral area

Laboratory investigations revealed undetectable follicle-stimulating hormone and luteinizing hormone (<0.1/<0.1 IU/L), an elevated estradiol [168 pmol/L, (normal range <60 pmol/L)], a suppressed thyroid-stimulating hormone [<0.01 mIU/L, (normal range: 0.73-4.09 mIU/L)] and elevated free T4 [20.1 pmol/L, (normal range: 10-17.6 pmol/L)]. Cortisol level was normal [214 nmol/L, (normal range: 14-458 nmol/L)]. An initial bone age assessment after the episode of vaginal bleeding, revealed a slight advancement (by 4 to 10 months). However, follow up radiography performed at 21 months of age revealed an advancement of 21 months, with a bone age of 42 months. Skeletal survey revealed evidence of lesions in the femur and humerus suggestive of polyostotic fibrous dysplasia (Figure 1). A routine abdominal ultrasound showed an incidental finding of a bi-lobed left adnexal mass suggestive of an ovarian cyst (Figure 2). Assessment of the renal tubular absorption rate of phosphate did not reveal evidence of FGF23 mediated phosphate wasting. However, upon retrospective chart review, it was noted that she had had hypophosphatemia requiring phosphate supplements for several months prior to her transplant. 

Written informed consent was obtained from the parents of the patient.

### Final Diagnosis

Given the constellation of findings of gonadotropin independent precocious puberty, mild hyperthyroidism, café-au-lait macules and the appearance of polyostotic fibrous dysplasia, a clinical diagnosis of MAS was made. The question of whether the previous hepatobiliary dysfunction was an early pathologic manifestation of MAS was raised. G-protein coupled receptor mutation analysis was completed on native liver tissue obtained at transplant which identified one of the typical pathogenic variants (c.602G>A.p.R201H) in exon 8 of the *GNAS1 (adenylate cyclase stimulatory G protein)* gene that has been associated with the MAS phenotype (1,2,3).

### Hospital Course

Over the next six months, the patient continued to develop recurrent episodes of vaginal bleeding with evidence of an advancing bone age. She was initiated on a trial of an aromatase inhibitor for management of precocious puberty. She sustained recurrent fractures of her femur and received a course of bisphosphonate therapy. She has required intermittent treatment with methimazole for thyroid over-activity. She is receiving ongoing clinical surveillance of bone lesions and screening for other comorbidities of MAS.

## Discussion

MAS is caused by activating somatic mutations within the *G protein *a* stimulatory subunit (GNAS)* gene (1). These mutations occur in the early post-zygotic period and the clinical presentation of patients will vary depending on the unique pattern of affected cells. Hepatic involvement has been described in some of the earliest case reports as an uncommon but early manifestation of MAS (1). One case series reported 16 patients with MAS and evidence of liver disease between 1937 and 1993 (2). Lumbroso et al (3) presented a case series of 113 patients with MAS, six of whom had evidence of cholestasis. Specific G-protein mutations have been identified in two case reports of patients with cholestasis and MAS (4,5). To our knowledge, our case is the first presentation of severe cholestasis with significant associated morbidity, ultimately leading to liver transplantation (6).

The underlying mechanism by which the constitutive activity of the G protein leads to cholestasis is unclear, however it has been suggested to play a role in bile metabolism (4). G protein coupled receptors play an important role in regulating intracellular signaling pathways in biliary epithelial cells. Interruption of normal signal transduction could impair cellular function, affecting bile formation and secretion by the cholangiocyte (4,7). Normalization of the liver function tests and complete resolution of the cholestasis does not always occur. However, existing descriptions of the natural history of the cholestasis suggest a benign course in most patients, with slow improvement and stabilization over time (4). In previously published case reports, where biochemical data are available, the liver enzymes are typically only mildly elevated (four to five-fold). The profound abnormalities seen in our patient (peak AST 33-fold elevation and peak ALT 35-fold elevation) are in marked contrast to these previous reports. With somatic mosaicism, the severity of the hepatic phenotype likely varies according to the number of cells affected by the mutation. This may account for the spectrum of liver disease described in the literature. Clinical presentation in our patient was affected by a number of secondary complications including failure to thrive, recurrent infections and fractures. Interestingly, following liver transplantation, many of these comorbidities resolved or improved significantly. 

Hepatobiliary lesions and hepatic adenomas have been identified in adult patients with MAS, further supporting the concept of persistent liver involvement as a pathologic feature of the syndrome (8). Less commonly, other gastrointestinal manifestations of MAS have been described, including intestinal polyps, pancreatitis and intra-ductal papillary mucinous neoplasms (4,8,9). Importantly, the authors advocate for consideration of radiographic screening of patients with MAS given the risk of malignancy associated with pancreatic intraductal papillary mucinous neoplasms, hepatic adenomas and choledochal cysts. This may be particularly relevant for patients with identified gastrointestinal manifestations.

The reported patient was started on letrozole for treatment of precocious puberty. Aromatase inhibitors have been associated with mild liver abnormalities among women taking it as an adjuvant treatment for breast cancer and rarely it has been associated with more significant hepatoxicity (10). Longitudinal follow up of a pediatric cohort of patients with MAS treated with letrozole for precocious puberty did not report on any hepatic side effects (11). However, it is unclear if any patients in this series had pre-existing hepatic disease. Given this patient’s history of liver transplantation, continued surveillance of liver function will be performed during her treatment course. This adverse reaction should also be considered for other patients with MAS and potential hepatic involvement.

The case reported here exhibits a severe phenotype of persistent cholestasis in MAS which has been previously considered to be a more benign feature. While this is a rare finding, we propose that MAS should be considered in the differential diagnosis of unexplained cholestasis. Occurring in the neonatal period, the presence of cholestatic liver disease may provide an early clue to the underlying diagnosis of MAS. In turn, prompt diagnosis may allow for screening, recognition and treatment of other endocrinopathies and bone disease.
